# Supplementary material for: Borna Disease Virus 1 Phosphoprotein Forms a Tetramer and Interacts with Host Factors Involved in DNA Double-Strand Break Repair and mRNA Processing
Source: Viruses. 2022 Oct 26;14(11):2358. doi: 10.3390/v14112358 (PMC9692295; doi:10.3390/v14112358)
Supplement: Supplementary file 1 [file viruses-14-02358-s001.zip › 20221013_supdata.pdf]

Supplementary data

Borna Disease Virus 1 Phosphoprotein Forms a Tetramer and Interacts with Host Factors Involved in DNA Double-Strand Break Repair and mRNA Processing

Nicolas Tarbouriech, Florian Chenavier, Junna Kawasaki, Kamel Bachiri, Jean-Marie Bourhis, Pierre Legrand, Lily Freslon, Estelle MN Laurent, Elsa Suberbielle, Rob WH Ruigrok, Keizo Tomonaga, Daniel Gonzalez-Dunia, Masayuki Horie, Etienne Coyaud and Thibaut Crépin

Experimental procedures

Generation of Bio-ID samples

BioID samples were prepared from Flp-In™ T-REx™ HEK293 expressing N- or C-terminal BirA*-flagged P protein or BirA*alone. Briefly, three independent replicates of two 150 cm^2 plates of sub-confluent (60 %) cells were incubated for 24 hours in complete medium supplemented with 1 $\mu g.mL^{-1}$ tetracycline (Sigma), 50 μM biotin (Thermo Fisher Scientific). Cells were collected and pelleted (300 g, 3 min), washed twice with PBS, and dried pellets were snap frozen. Each cell pellet was resuspended in 5 mL of lysis buffer (50 mM Tris-HCl pH 7.5, 150 mM NaCl, 1 mM EDTA, 1 mM EGTA, 1 % Triton X-100, 0.1 % SDS, 1:500 protease inhibitor cocktail (Sigma-Aldrich), 1:1,000 Turbonuclease (BPS Bioscience) and incubated on an end-over-end rotator at 4°C for 1 hour, briefly sonicated to disrupt any visible aggregates, then centrifuged at 45,000 *g* for 30 min at 4°C. Supernatant was transferred to a fresh 15 mL conical tube. 30 μL of packed, pre-equilibrated Streptavidin Ultralink Resin (Pierce) were added and the mixture incubated for 3 hours at 4°C with rotation. Beads were pelleted by centrifugation at 300 *g* for 2 min and transferred with 1 mL of lysis buffer to a fresh eppendorf tube. Beads were washed once with 1 mL of lysis buffer and twice with 1 mL of 50 mM ammonium bicarbonate pH 8.3, then transferred in ammonium bicarbonate to a fresh centrifuge tube and washed two more times with 1 mL of ammonium bicarbonate buffer. Tryptic digestion was performed by incubating the beads with 1 μg MS-grade TPCK trypsin (Promega) dissolved in 200 μL of 50 mM ammonium bicarbonate pH 8.3 overnight at 37°C. The following morning, 0.5 μg MS-grade TPCK trypsin was added to the beads and incubated 2 additional hours at 37°C. Following centrifugation at 2,000 g for 2 min, the supernatant was collected and transferred to a fresh eppendorf tube. Two additional washes were performed with 150 μL of 50 mM ammonium bicarbonate and pooled with the first eluate. The sample was lyophilised and resuspended in buffer A (2 % CAN, 0.1 % formic acid). $1/4^{th}$ of each sample was analysed per mass spectrometer run.

BioID data acquisition

MS samples were prepared from 20 control samples and three biological replicates of the P bait protein fused either with a N-terminal or a C-terminal BirA*Flag epitope tag and analysed on a Thermo Q-Exactive mass spectrometer. Samples were separated by online reversed-phase chromatography using a Thermo Scientific Easy-nLC1000 system equipped with a Proxeon trap column (75 μm ID x 2 cm, 3 μm; Thermo Scientific,) and a C18 packed-tip column (Acclaim PepMap, 75 μm ID x 50 cm, 2 μm; Thermo Scientific). The digested peptides were separated using an increasing amount of acetonitrile in 0.1 % formic acid from 2 to 30 % for 2 hours at a flow rate of 300 $nL.min^{-1}$. A voltage of 2.4 kV was applied by the liquid junction to electrospray the eluent using the nanospray source. A high-resolution mass spectrometer Q-Exactive™ Thermo Scientific™ was coupled to the chromatography system to acquire the 10 most intense ions of MS1 analysis (Top 10) in data dependent mode. The MS analyses were performed in positive mode at a resolving power of 70,000 FWHM, using an automatic gain control target of 3 x 10^6, the default charge state was set at 2 and a maximum injection time at 120 ms. For full scan MS, the scan range was set between m/z 300 to 1600. For ddMS2, the scan range was between m/z 200 to 2000, 1 microscan was acquired at 17,500 FWHM, an AGC was set at 5 x 10^4 ions and an isolation window of m/z 4,0 was used.

BioID data analysis

The proteins were identified by comparing all MS/MS data with the *Homo sapiens* proteome database (Uniprot, release March 2020, Canonical+Isoforms, comprising 42,360 entries + BirA* and P protein sequences added manually), using the MaxQuant software (version 1.5.8.3). The digestion parameters were defined using trypsin with 2 maximum missed cleavages. The oxidation of methionine and N-terminal protein acetylation were defined as variable modifications. The Label-free quantification (LFQ) was done keeping

the default parameters of the software. As for initial mass tolerance, 6 ppm was selected for MS mode, and 20 ppm was set for fragmentation data to match MS/MS tolerance. The identification parameters of the proteins and peptides were performed with a false discovery rate (FDR) at 1%, and a minimum of two unique peptides per protein. The LFQ values from the twenty control runs (regrouping FlagBirA* and BirA*Flag alone samples, from stably transfected cell lines) were collapsed to the three highest values for each given ID (Table S3;b). These three values were defined as the control group for comparison with viral bait protein biological triplicates. The statistical analysis was done by Perseus software (version 1.6.2.3). Briefly, the LFQ intensity of each sample was downloaded in Perseus and the data matrix was filtered by removing the potential contaminants, reverse and only identified by site. The data were then transformed using the log2(x) function. Before statistical analysis, two groups (N-ter, C-ter BirA*-tagged P) were defined with three replicates per group. Only preys with detected values in all three replicates of a given viral bait protein were kept for further analysis. Missing values were then replaced from normal distribution separately for each column. Two-sample Student's T-test was then performed comparing all three biological replicates of each bait and condition against the three highest intensities control runs. High confidence proximal interactors were defined by permutation-based FDR with a cut-off of 0.01. Perseus output with all experimental values is reported in Table S3. This matrix shows the average log2 fold change against control and the corresponding q-values for each bait. Word cloud was performed on corresponding GO terms using SRplot online software (http://www.bioinformatics.com.cn/) and VolcaNoseR [1].

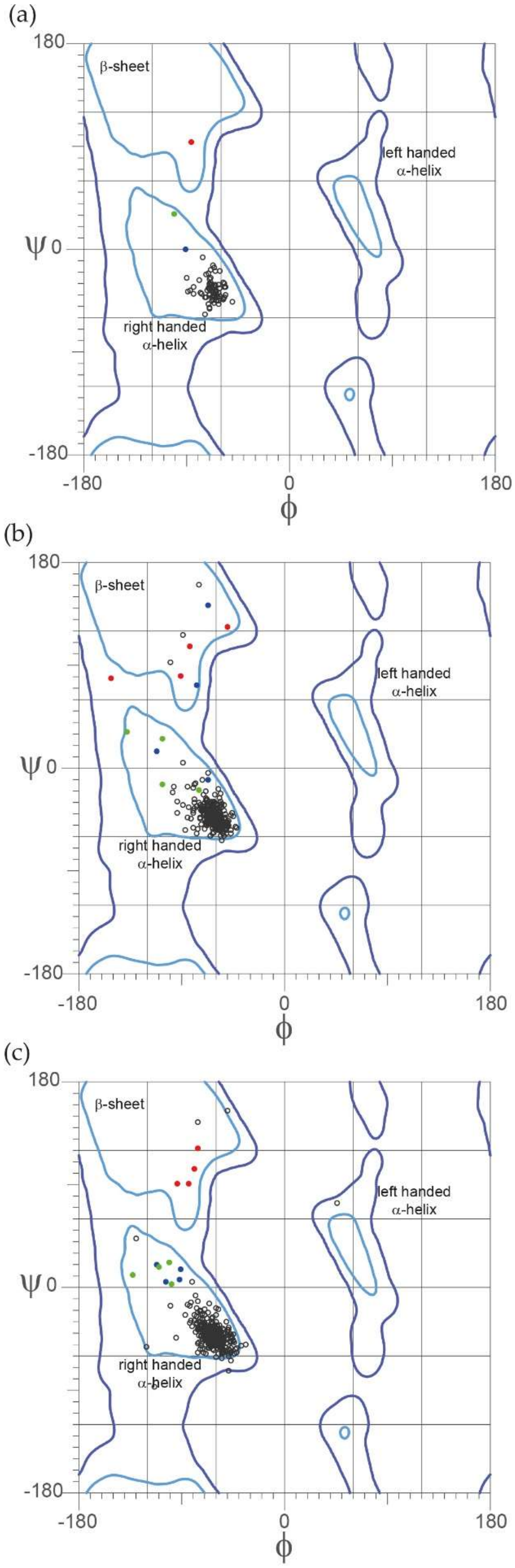

Figure S1. Ramachandran plot of the three PoD X-ray structures. The figure shows the energetically allowed regions for the backbone dihedral angles ψ against φ of amino acid residues in (a) BoDV-1, (b) MuBV-1 and (c) GaVV-1 PoD; position one (C125, T125 and L130), two (D126, D126 and D131) and three (H127, Y127 and Y132) of the kink are coloured in blue, red and green respectively; the plots have been extracted from the MolProbity [2] analysis.

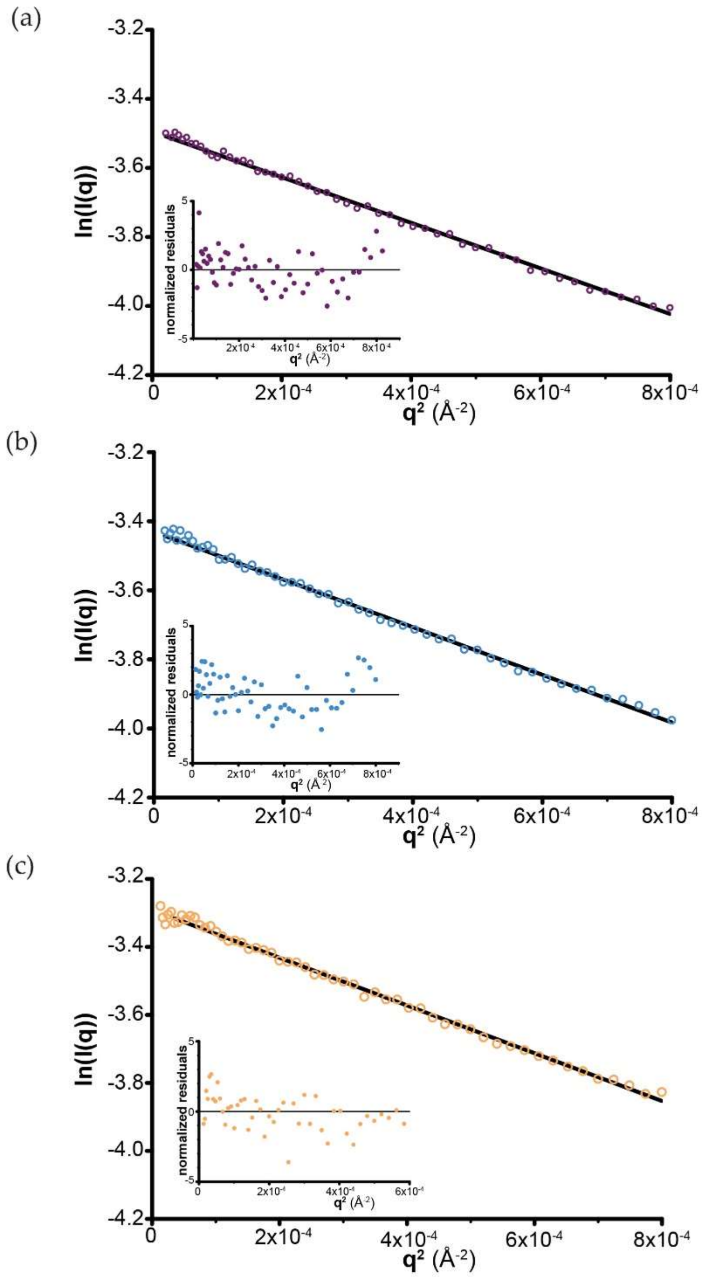

Figure S2. Guinier analysis of the Pod SAXS analysis. The Guinier extrapolation of (a) BoDV-1, (b) MuBV-1 and (c) GaVV-1 POD are shown with the smallest plots corresponding to their respective normalised residual.

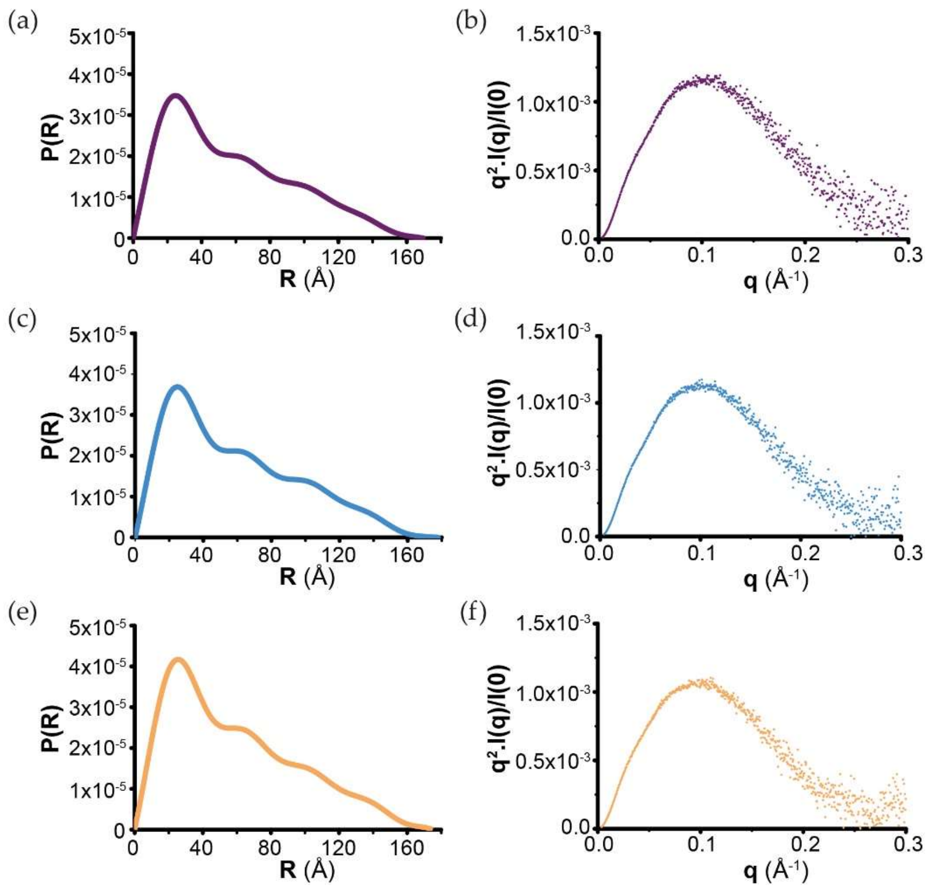

Figure S3. P(R) functions and Krakty plots of the POD SAXS analysis. The figure corresponds to representations of (a-b) BoDV-1, (c-d) MuBV-1 and (e-f) GaVV-1 POD respectively.

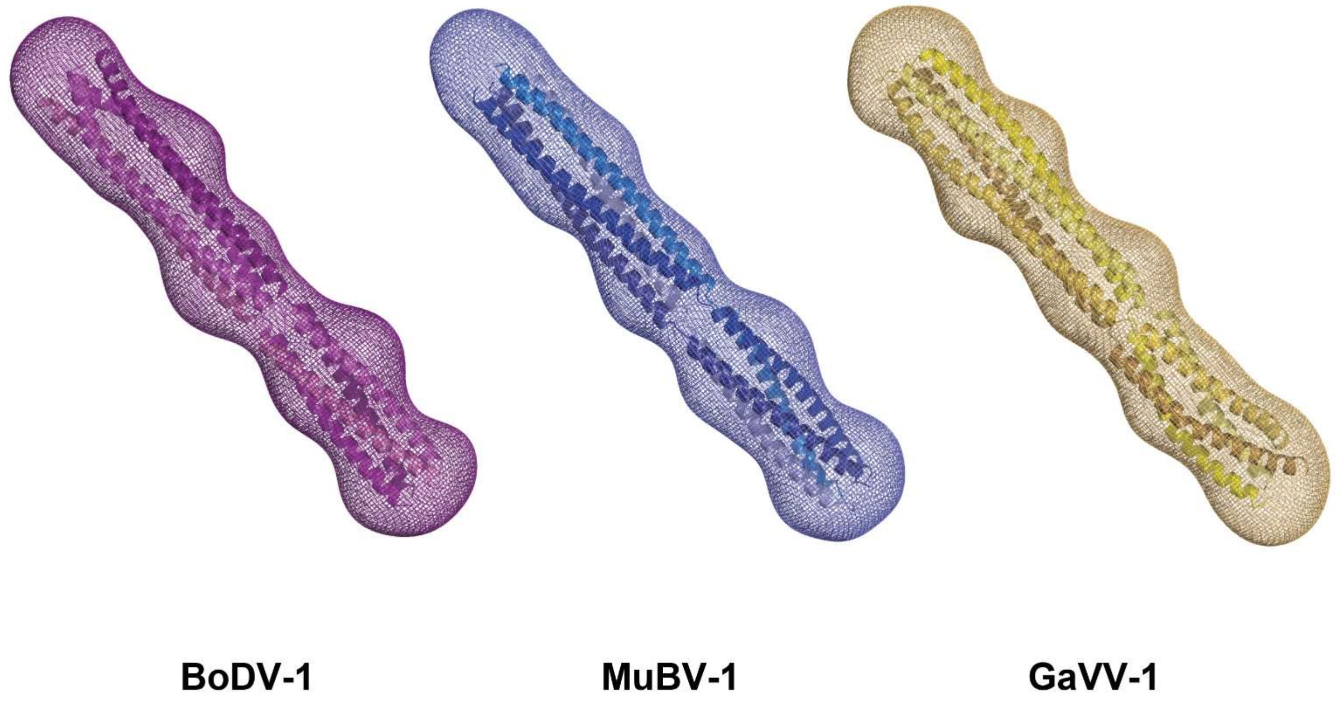

Figure S4. Orthobornaviral POD SAXS envelops. The figure compares the three mean envelops obtained from DAMAVER with the corresponding X-ray structures docked inside.

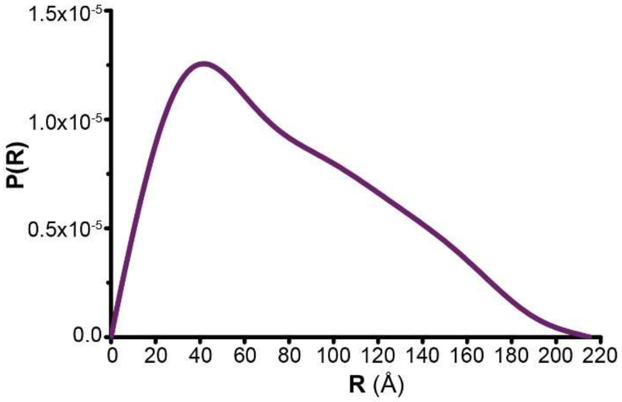

Figure S5. P_{FULL} P(r) function.

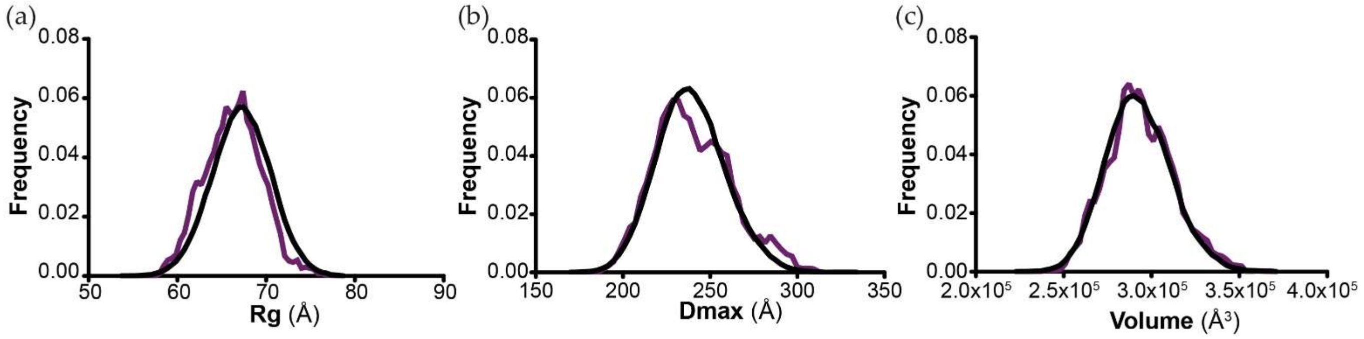

Figure S6. Validity of the six PFULL models presented on Figure 2.d. The figures compare the mean (a) Rg, (b) Dmax and (c) volume of the six models of the figure (purple curve) and the 10,000 models (black curve) generated during the PFULL modelling procedure based on EOM.

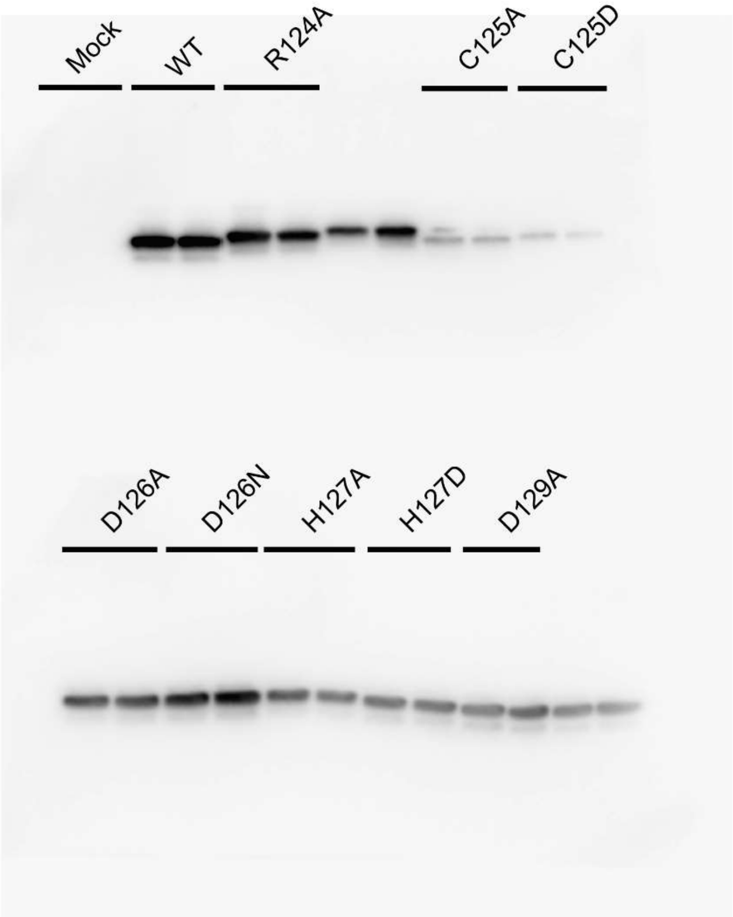

Figure S7. Detection of BoDV-1 P mutants. The figure presents the whole western-blot shown on Figure 3.

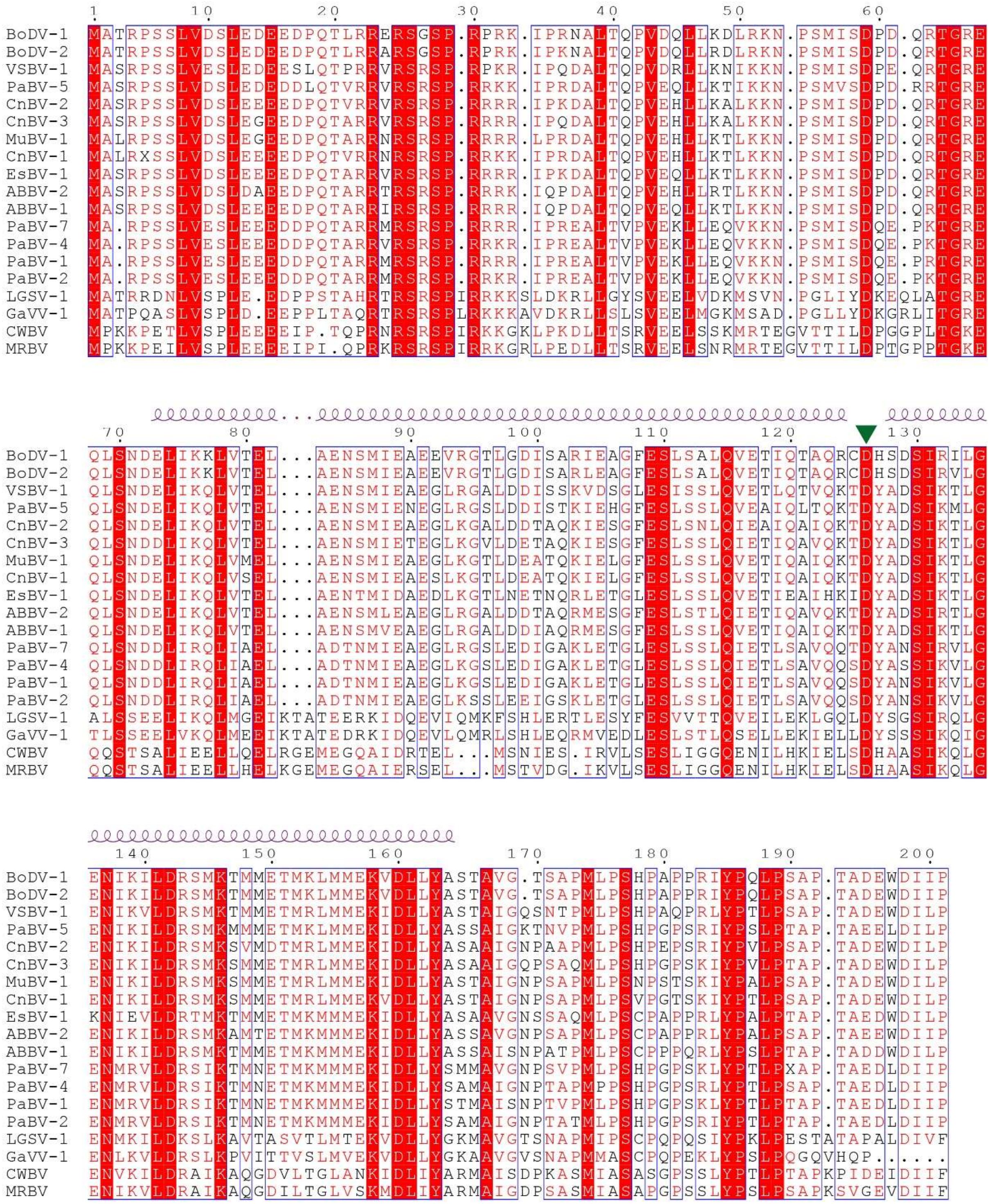

Figure S8. Sequence alignment of P from all representative viruses of the genus *Orthobornavirus*. BoDV-1, Borna disease virus 1 (AJ311522); BoDV-2, Borna disease virus 2 (AJ311524); VSBV-1, variegated squirrel bornavirus 1 (LN713680); PaBV-5, parrot bornavirus 5 (GU249595); CnBV-2, canary bornavirus 2 (KC464478); CnBV-3, canary bornavirus 3 (KC595273); MuBV-1, munia bornavirus 1; CnBV-1, canary bornavirus 1 (KC464471); EsBV-1, estrildid finch bornavirus 1 (KF680099); ABBV-2, aquatic bird bornavirus 2 (KJ756399); ABBV-1, aquatic bird bornavirus 1 (KF578398); PaBV-7, parrot bornavirus 7 (JX065210); PaBV-4, parrot bornavirus 4 (JX065209); PaBV-1, parrot bornavirus 1 (GU249595); PaBV-2, parrot bornavirus 2 (FJ620690); LGSV-1, loveridges garter snake virus 1 (KM114265); GaVV-1, Gaboon viper virus 1 (AB714966); CWBV, Caribbean watersnake bornavirus (BK014571); MRBV, Mexican black-tailed rattlesnake bornavirus (BK014572); the green triangle indicates the position of the conserved aspartate residue within the kink; the sequences were aligned using Clustal W [3] and manually adjusted; the figure was drawn with ESPript [4].

(a)

(b)

(c)

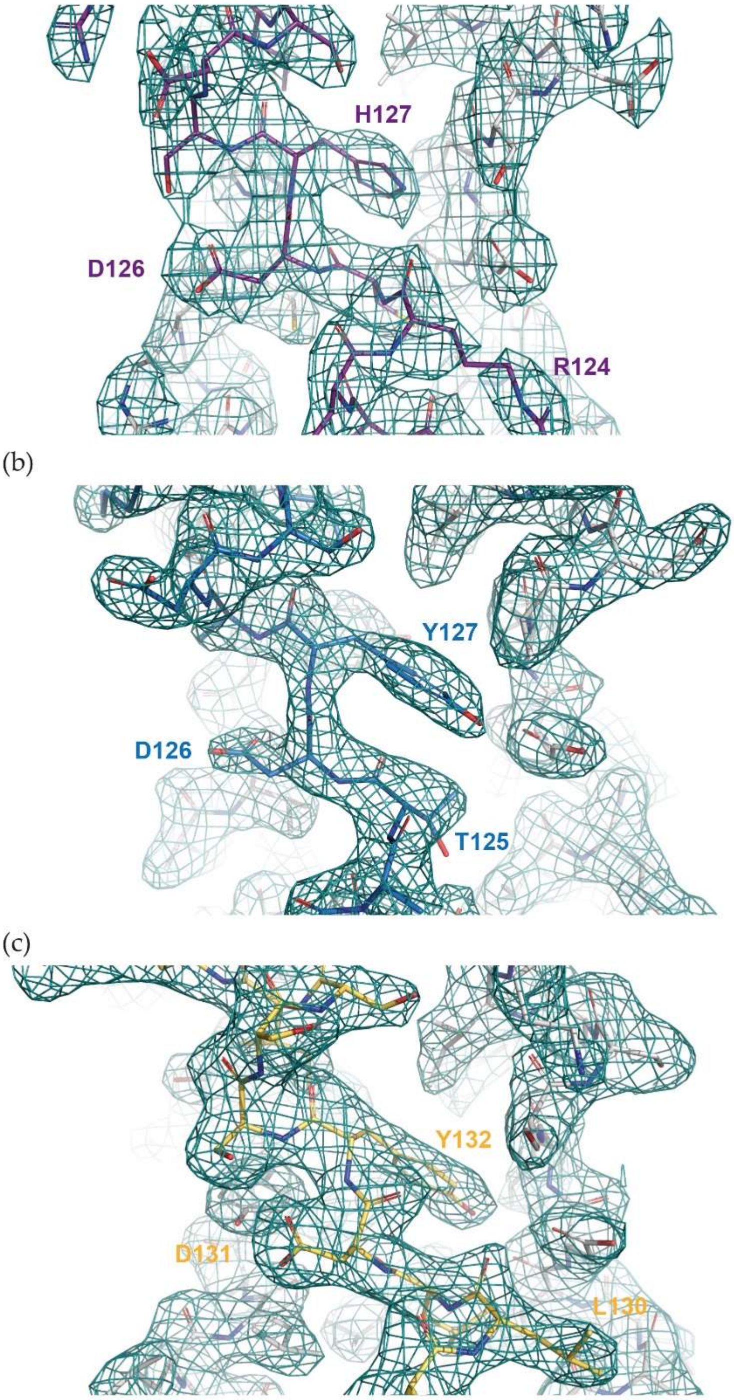

Figure S9. Detail of the electron density maps. The figure shows the electron density of (a) BoDV-1, (b) MuBV-1 and (c) GaVV-1 breaks.

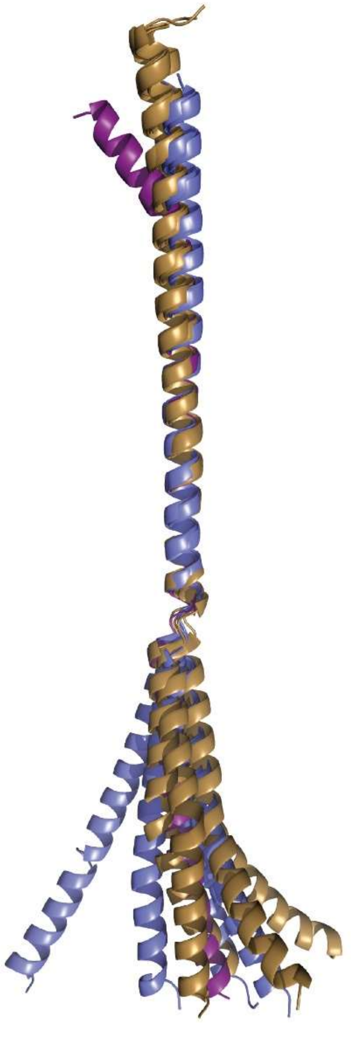

Figure S10. Orthobornaviral P_{OD} flexibility. The figure shows the superimposition based on the first α-helix of all individual chains from the BoDV-1 (purple), MuBV-1 (blue) and GaVV-1 (dark yellow) crystal structures.

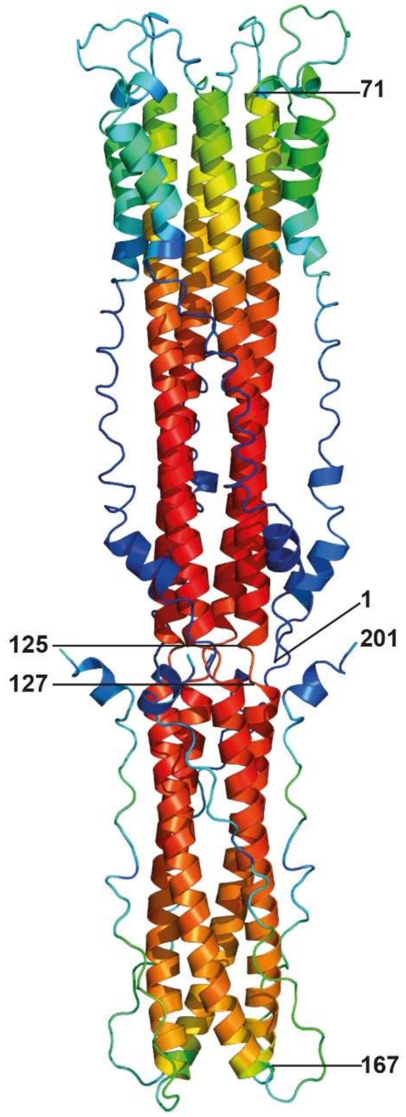

Figure S11. BoDV-1 P structure prediction by AlphaFold2. The model is coloured according to the predicted local-distance difference test (pLDDT) from the lower (blue) to the higher (red) confidence. The model is predicted with a break in the central domain, from residue 125 to 127.

Table S1. X-ray data-collection and refinement parameters.

	BoDV-1 native	MuBV-1 native	GaVV-1 native	GaVV-1 SeMet
Data collection				
Instrument	ESRF - ID29	SOLEIL - PX1	SOLEIL - PX1	SOLEIL - PX2
Wavelength (Å)	0.97717	0.978565	0.978565	0.98919
Space group	$P42_12$	$P2_1$	$P2_12_12$	$P2_12_12$
a,b,c (Å)	35.3, 35.3, 166.5	70.2, 42.7, 72.2	81.8, 82.5, 89.0	80.8, 81.7, 89.5
α,β,γ (°)	90, 90, 90	90, 92.36, 90	90, 90, 90	90, 90, 90
Resolution range [1] *(Å)*	55-2.75 (2.82-2.75)	49-2.15 (2.22-2.15)	49-2.40 (2.46-2.40)	48-2.79 (2.87-2.79)
R_{merge} [1,2]	0.258 (3.26)	0.052 (0.67)	0.114 (2.946)	0.106 (2.124)
R_{pim} [1,2]	0.108 (1.357)	0.037 (0.471)	0.046 (1.168)	0.049 (0.99)
$I/\sigma I$ [1,2]	5.0 (0.6)	15.2 (2.0)	11.4 (1.0)	10.2 (1.0)
Completeness [1,2] *(%)*	99.9 (99.7)	99.9 (100)	99.6 (99.4)	99.2 (89.3)
Total reflections [1,2]	35,192 (2,520)	128,423 (11,377)	324,542 (24,198)	155,314 (9,913)
Unique reflections [1,2]	3,173 (214)	23,663 (2,043)	24,108 (1,742)	15,170 (988)
Multiplicity [1,2]	11.1 (11.8)	5.4 (5.6)	13.5 (13.9)	10.2 (10.0)
CC(1/2) [1,2]	0.998 (0.601)	0.998 (0.808)	0.999 (0.620)	0.999 (0.657)
Anomalous completeness [1,2] *(%)*	-	-	-	98.7 (86.5)
Anomalous multiplicity [1,2]	-	-	-	5.4 (5.3)
DelAnom correlation [1,2]	-	-	-	0.674 (0.066)
Refinement [3]				
No. reflections	2,642	22,567	18,296	-
R_{work} / R_{free}	24.8 / 34.6	21.8 / 30.8	22.9 / 31.5	-
No. non-H atoms	748	3,137	3,375	-
No. water	9	114	156	-
R.m.s. deviations				
Bond length (Å)	0.009	0.007	0.007	-
Bond angle (°)	0.97	1.446	1.367	-
Ramachadran statistic [3]				
Favoured (%)	98.9	99.2	96.7	-
Allowed (%)	1.1	0.8	2.5	-
Outlier (%)	0	0	0.8	-
Molprobity scores [3]				
Score	1.47	1.55	2.73	-
All-atom clashscore	2.66	10.74	11.65	-
PDB code	8B8A	8B8B	8B8D	-

[1] Values in parentheses are for the highest-resolution shell.
[2] As reported by autoPROC and STARANISO.
[3] as reported by MolProbity [2].

Table S2. SAXS data-collection and scattering-derived parameters.

	BoDV-1 P_{OD}	MuBV-1 P_{OD}	GaVV-1 P_{OD}	BoDV-1 P_{Full}
Data collection parameters				
Instrument	SOLEIL - SWING			
Energy (keV)	12.000			
Detector	EigerX-4M			
Detector distance (m)	2			
Exposure (s per image)	1			
q range (Å^{-1})	0.0036 - 0.54			
Column	S200inc 5/150 GL			
Flow rate (mL.min^{-1})	0.3			
Sample concentrations (mg.mL^{-1})	15.1	12.4	12.9	4.6
Injection volume (μL)	8	8	15	50
Temperature (K)	293	293	293	293
Guinier Analysis				
I(0) (A.U.)	0.030 ± 6.10^{-5}	0.032 ± 4.10^{-5}	0.037 ± 7.10^{-5}	0.017 ± 1.10^{-4}
Rg (Å)	44.5 ± 0.14	45.5 ± 0.14	45.9 ± 0.1	61.3 ± 0.6
qrgmin	0.16	0.16	0.17	0.3
qrgmax	1.3	1.28	1.3	1.29
P(R) analysis [1]				
Dmax (Å)	170 ± 9	178 ± 8	176 ± 10	215 ± 10
Molecular weight [2]				
Theoretical Mw (kDa) [3]	47	49.6	51.6	100
Measured Mw (kDa)	40	41.5	45.5	90
Deviation (%)	15	16	12	10
DAMMIN [4]				
q range (Å^{-1})	0.2	0.2	0.2	
symmetry	P4	P4	P4	
Nbr of average models	15	15	15	
χ^2	1.2	1.2	1.2	
EOM [4,5]				
Nbr of unique models				6
χ^2				1.0
SASBDB code	SASDQQ5	SASDQR5	SASDQS5	SASDQP5

[1] from GNOM [5] and BIFT [6].
[2] from volume of correlation.
[3] from sequence.
[4] default parameters
[5] 10,000 full atoms models

References

1. Goedhart, J.; Luijsterburg, M. S., VolcaNoseR is a web app for creating, exploring, labeling and sharing volcano plots. *Sci Rep* **2020,** 10, (1), 20560.
2. Williams, C. J.; Headd, J. J.; Moriarty, N. W.; Prisant, M. G.; Videau, L. L.; Deis, L. N.; Verma, V.; Keedy, D. A.; Hintze, B. J.; Chen, V. B.; Jain, S.; Lewis, S. M.; Arendall III, W. B.; Snoeyink, J.; Adams, P. D.; Lovell, S. C.; Richardson, J. S.; Richardson, D. C., MolProbity: more and better reference data for improved all-atom structure validation. **2018,** 27, (1), 293-315.
3. Larkin, M. A.; Blackshields, G.; Brown, N. P.; Chenna, R.; McGettigan, P. A.; McWilliam, H.; Valentin, F.; Wallace, I. M.; Wilm, A.; Lopez, R.; Thompson, J. D.; Gibson, T. J.; Higgins, D. G., Clustal W and Clustal X version 2.0. *Bioinformatics* **2007,** 23, (21), 2947-8.
4. Gouet, P.; Courcelle, E.; Stuart, D. I.; Metoz, F., ESPript: analysis of multiple sequence alignments in PostScript. *Bioinformatics* **1999,** 15, (4), 305-8.
5. Svergun, D., Determination of the regularization parameter in indirect-transform methods using perceptual criteria. *Journal of Applied Crystallography* **1992,** 25, (4), 495-503.
6. Hansen, S., Bayesian estimation of hyperparameters for indirect Fourier transformation in small-angle scattering. *Journal of Applied Crystallography* **2000,** 33, (6), 1415-1421.
